# An interesting electrocardiogram caused by lead reversal

**DOI:** 10.1186/s12872-024-03875-2

**Published:** 2024-04-20

**Authors:** Changjun Li, Nan Wang, Qinghua Chang, Dianzhu Pan

**Affiliations:** 1 Department of Gerontology, The First Affiliated Hospital of Jinzhou Medical University, Renmin Street, Jinzhou, 121000 Liaoning Province China; 2 Department of Cardiology, The First Affiliated Hospital of Jinzhou Medical University, Renmin Street, Jinzhou, 121000 Liaoning Province China; 3 Department of Respiratory, The First Affiliated Hospital of Jinzhou Medical University, Renmin Street, Jinzhou, 121000 Liaoning Province China

**Keywords:** Limb-lead reversals, Electrocardiogram, Mirror-image dextrocardia, Acquired dextrocardia, Left atrial rhythm

## Abstract

**Background:**

During normal sinus rhythm, atrial depolarization is conducted from right atrium to left atrium through Bachmann’s bundle, and a normal P wave axis which is measured on the frontal plane is between 0º and + 75º. The change of P wave polarity is helpful for the analysis of origin point.

**Case presentation:**

We report a patient with negative P wave in lead I. The characteristics of QRS complex in leads V_1_ to V_6_ are helpful to preliminarily differential diagnosis. The 12-lead electrocardiogram (ECG) with correct limb leads (right arm-left arm) placement shows sinus rhythm with complete right bundle branch block (RBBB).

**Conclusions:**

The change of P wave polarity as well as characteristics of QRS complex can help identify limb-lead reversals.

## Background

Limb-lead reversals on electrocardiogram (ECG) have an estimated frequency of 0.4% in the outpatient setting to as high as 4% in the intensive care unit [1]. Left arm and left leg lead reversals can lead to the misidentification of inferior wall ST-segment elevation myocardial infarction (STEMI) as lateral STEMI [2]. Promptly identifying lead reversal is important to avoid diagnostic and treatment delays of any clinical situation. We report a patient with negative P wave in lead I as a result of right arm-left arm lead reversal.

## Case presentation

**Fig. 1:**
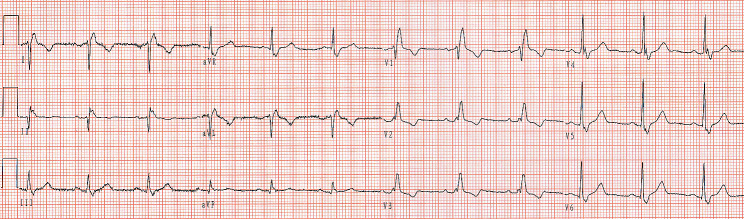
Patient’s initial 12-lead ECG

**Fig. 2:**
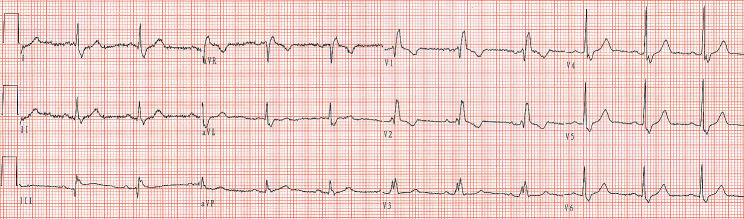
The 12-lead ECG with correct limb leads placement shows sinus rhythm with complete right bundle branch block

A man in his 60s presented to our hospital with complaints of poor activity of the left limbs accompanying barylalia for the past 6 days. His medical history included hypertension for which he took amlodipine. He had no other medical history, including diabetes mellitus and coronary heart disease. On examination, his blood pressure was 157/80 mmHg, with a heart rate of 78/min and respiratory rate of 20/min. Transthoracic echocardiography showed discordant left ventricular myocardial motion and left atrial enlargement (40 mm). His initial ECG is shown in Fig. 1, which demonstrates a wide QRS complex (140 milliseconds) with rsR′ pattern in lead V_1_ and Rs pattern in lead V_5_ (S wave of greater duration than 40 ms), indicating a complete right bundle branch block (RBBB). P wave morphology shows negative deflection in lead I, biphasic deflection in lead II and positive polarity in lead aVR. Besides, the P-QRS-T polarity in lead aVR is identical to that seen in leads V_5_ and V_6_. These features aforementioned should raise the suspicion of the presence of limb electrode reversal. The 12-lead ECG with correct limb leads (right arm-left arm) placement shows sinus rhythm with complete RBBB (Fig. 2).

## Discussion and conclusion

The P wave on the ECG represents atrial tissue activation. During normal sinus rhythm, atrial depolarization is conducted from right atrium to left atrium through Bachmann’s bundle, and a normal P wave axis which is measured on the frontal plane is between 0º and + 75º [3]. Therefore, the atrial depolarization vector projection on the lead II and aVR results in positive polarity of lead II and negative polarity of lead aVR. Besides, P wave morphology shows positive deflection in lead I. When the limb and precordial leads are placed in their standard location, the aVR and V_5_ or V_6_ leads are in the same alignment but have deflections in the opposite polarity [4].

In our case, P wave morphology shows biphasic deflection (initially positive followed by negative deflections) in lead II and positive polarity in lead aVR, indicating that it isn’t likely the result of normal sinus rhythm. P wave morphology shows negative deflection in lead I, indicating that it is likely the result of conduction from the left atrium to the right atrium. The 5 most likely causes of depolarization sequence are the mirror-image dextrocardia, acquired dextrocardia, left atrial rhythm, the right arm-left leg lead reversal and the right arm-left arm lead reversal [5, 6]. The characteristics of QRS complex in leads V_1_ to V_6_ are helpful to preliminarily differential diagnosis. Figure 1 shows a RBBB morphology in chest leads. There is a progressive increase in R-wave amplitude from leads V_1_ to V_6_, which isn’t favor of mirror-image dextrocardia. Transthoracic echocardiography also didn’t show mirror-image dextrocardia. The causes of acquired dextrocardia (dextroposition) include lung, pleural, and diaphragm lesions, such as right pneumonectomy, left pneumothorax, and left lung hypoplasia, which can induce negative P wave in lead I. Recently, Tsai et al. [6] reported one patient with inverted P and T waves in lead I and aVL, and dominant R wave in lead V_1_ in acquired dextrocardia. Our patient denied any above medical history and intra-abdominal or thoracic symptoms. The echocardiography showed left atrial enlargement, which can cause left atrial rhythm. If this was an example of a left atrial rhythm, QRS complex morphology is consistent with conduction to the ventricles through the normal His-Purkinje atrioventricular conduction system (normal QRS complex or BBB). The aVR and V_5_ leads displayed identical polarity deflections, excluding left atrial rhythm [4]. At this time, we should suspect the presence of abnormal placement of limb leads. Besides, in a 2021 study, Littmann [7] proposed that lead V_1_ can mimic aVR. The V_1_ and aVR discordance also should raise the suspicion of the presence of electrode reversal. Right arm-left leg reversal leads is uncommon. Right arm-left leg reversal produces highly abnormal-looking limb leads, with leads I, II, III, and aVF being negative and aVR being upright, mimicking inferior myocardial infarction. The aVL is unchangeable. In our case, P waves in leads III and aVF are positive and the QRS complex morphology in lead aVL is inconsistency with lead V_5_, therefore it is highly unlikely to have a right arm-left leg reversal. Right arm-left arm reversal lead is common. In a patient with a right arm-left arm lead reversal, the P-QRS-T in lead I should be flipped, the pattern of lead aVR resembles a normal aVL, and lead II resembles a normal lead III [8]. The initial ECG (Fig. 1) corrected according to the aforementioned features was consistent with typical RBBB. The repeat ECG in Fig. 2 after correcting right arm-left arm lead connection shows sinus rhythm with RBBB, confirming right arm-left arm lead reversal on initial ECG.

The ECG is a simple and inexpensive tool which can be recorded by many different types of health personnel, including physician doctors, ECG technicians, nurses from a variety of hospital units. The value of the ECG depends upon the accuracy of how it is obtained. When right arm-left arm lead reversal is not complicated with arrhythmia, ECG features are typical and easy to diagnose. If combined with other abnormal ECG changes, such as the RBBB in this patient, electrocardiographic manifestations are diverse and atypical. Recognizing right arm-left arm lead reversal in patients with RBBB is an important and intellectually rewarding skill. Close evaluation of the lead characteristics of P-QRS-T changes such as lead I and chest leads may provide clues in diagnosing right arm-left arm lead reversal and its differential diagnosis. Besides, it is important to identify anatomic landmarks and to clear up any other misconceptions. Finally, a quality improvement program should monitor the incidence of common errors such as right arm-left arm lead wire reversal and provide retraining when indicated.

## Data Availability

All relevant data supporting the conclusions of this article is included within the article.

## Abbreviations

ECG: Electrocardiogram
RBBB: Right bundle branch block
STEMI: ST-segment elevation myocardial infarction
